# Comment on “Impact of Chronic Kidney Disease Severity on Clinical Outcomes After Drug‐Eluting Stent Implantation: A Propensity Score–Matched Analysis”

**DOI:** 10.1002/clc.70499

**Published:** 2026-11-02

**Authors:** Mahendra Singh, Sung Soo. Han

**Affiliations:** ^1^ Specialization: Drug Delivery Nanomedicine, Hydrogels, Wound Healing Dehradun India; ^2^ Specialization: Smart Polymers, 3D Bioprinting, Hydrogels, Tissue Engineering School of Chemical Engineering, Major in Polymers and Biomaterials Yeungnam University Gyeongsan‐si Republic of Korea

**Keywords:** cardiovascular risk, chronic kidney disease, clinical outcomes, coronary intervention, drug‐eluting stents, estimated glomerular filtration rate, kidney function, major adverse cardiovascular events, propensity score matching, stent implantation

Dear Editor,

Zhu and Jin [1] looked at 55 pairs of patients. They checked if their estimated glomerular filtration rate (eGFR) was lower than 60 mL/min/1.73 m^2^ after having a drug‐eluting stent put in. They found that people with eGFR had a hazard ratio of 3.20 for bad heart events (MACE). It is important to study people with kidney problems. These people are not often in stent studies. Local information can help. Some parts of the study make it hard to know what the results really mean about how bad chronic kidney disease's.

The title and the conclusions talk about the severity of CKD. The study only looked at two groups. If you check the limitations, the rates of heart events were 27.3%, 30.0%, and 27.3% for stages G3a, G3b, and G4. There was no pattern when eGFR was below 60. This means using CKD severity to predict risk might not be right. It also makes you wonder who was not matched. Nineteen of the 74 patients with eGFR below 60 had no match. These patients were probably older and more anemic. They had the eGFR. So the matched results might not show what happens to people with kidney disease.

The model they used includes things that're partly because of kidney disease [2]. Anemia and heart failure happen because of kidney function. Matching on hemoglobin made a group that had a hemoglobin 9 g/L lower than people with good kidney function. When you adjust for things that come from kidney disease to outcomes you change the question you are asking. This can change the answer in either direction. A model that only uses things before kidney disease or a mediation analysis would make the “independent” link easier to understand.

The time they watched patients is also a problem. Patients were added from January 2020 to January 2025. The average time they looked at was 13.4 months with a range of 11.4 to 16.8 months. People who got treatment early in this time should have been watched for years. The paper does not say if the study ended at a fixed time or stopped when they could not find records. Taking out people who were not found before any event might have removed some people on purpose [3].

How they found kidney issues might be different between groups. A drop in eGFR by 30% or more needs blood tests. People who already have eGFR are more likely to get these tests. So some of the increase in kidney problems might be from checking, not from faster damage.

The sensitivity analysis is shown as support for the findings. The numbers changed a lot. The hazard ratio for MACE went up from 3.20 to 4.43. For kidney injury from contrast, it went up from 3.52 to 7.06. For bleeding, it went up from 1.28 to 3.10. These big changes mean the results depend a lot on how they did the model. In this group, giving all these numbers would be more open. The summary would be better if they included them [4].

Low eGFR is a known sign of risk after having a stent. Figuring out if the risk really gets worse with severe kidney disease and how matching and watching over time change the results would make the findings more helpful for doctors.

Reduced eGFR is a well‐established marker of risk after stenting [5]. Showing whether risk truly rises with severity, and how matching and follow‐up shaped the estimate, would make these findings more useful for clinicians.

## Author Contributions

M. S. and S. S. H. contributed to conceptualization, literature review, critical analysis, manuscript drafting, critical revision, and final approval of the manuscript. Both authors reviewed and approved the final version of the manuscript.

## Funding

The authors have nothing to report.

## Ethics Statement

The authors have nothing to report.

## Consent

The authors have nothing to report.

## Conflicts of Interest

The authors declare no conflicts of interest.

## Generative AI Statement

The authors used Claude (Anthropic) to draft and edit the text. The authors reviewed and edited the content and take full responsibility for it.

## Data Availability

Data sharing not applicable to this article as no datasets were generated or analyzed during the current study.
